# Evaluating Low HER2 Status in Invasive Breast Carcinoma via HER2 Immunohistochemistry, with HER2 FISH Correlation: A Cohort of 112 Patients

**DOI:** 10.1155/2023/9725647

**Published:** 2023-08-25

**Authors:** Gokce Deniz Ardor, Miglena K. Komforti, Helena Hanna, Onur Ibanoglu, Abigail Lochala, Aziza Nassar

**Affiliations:** ^1^Department of Laboratory Medicine and Pathology, Mayo Clinic College of Medicine and Science, 4500 Pablo Road, South Jacksonville, Florida 32224, USA; ^2^Department of Biomedical Sciences, University of South Florida, 4202 E Fowler Ave, Tampa, FL 33620, USA; ^3^Division of Transplant Surgery, Mayo Clinic College of Medicine and Science, 4500 Pablo Road, South Jacksonville, Florida 32224, USA; ^4^Arkansas Tech University, 215 W O St, Russellville, AR 72801, USA

## Abstract

**Introduction:**

Recent trials demonstrated clinically significant benefits in HER2-nonamplified breast cancer with HER2-low expression using novel anti-HER2 antibody-drug conjugates. Thus, HER2-low breast cancer was proposed as a separate diagnostic entity. Herein, we reclassify HER2-negative cancers according to the new HER2-low category using a modified system and further investigate HER2-very-low expression.

**Methods:**

114 HER2 immunohistochemistry (IHC)-negative invasive breast tumors were identified from the pathology database of Mayo Clinic, Jacksonville, FL, between January 2019 and August 2022. Two blinded breast pathologists (BP) independently rescored HER2 IHC slides at 200x and 400x magnification. Discordant cases between the two BPs were rescored together. The most recent 2018 ASCO/CAP HER2 scoring criteria were used. HER2 (0) was subdivided into HER2 (absent) and HER2 (very low). HER2 FISH testing was performed in all cases.

**Results:**

The cohort comprised of 38 (33.3%) HER2 (0) and 76 (66.7%) HER2 (1+) tumors. The first round of rescoring at 200x and 400x magnification resulted in 17 (14.9%) HER2 (absent), 31 (27.2%) HER2 (very low), and 64 (56.2%) HER2 (1+) and 2 (1.8%) HER2 (2+) tumors by BP1 and 20 (17.5%) HER2 (absent), 33 (28.9%) HER2 (very low), and 61 (53.5%) HER2 (1+) tumors by BP2. The combined final rescoring by BP1 and BP2 was as follows: 15 (13.2%) HER2 (absent), 35 (30.7%) HER2 (very low), 63 (55.3%) HER2 (1+), and 1 (0.9%) HER2 (2+) cases. A comparison of the first round of rescoring between two BPs showed substantial agreement with Cohen's kappa value of 0.67. Both comparisons of first rescoring by BP1 and by BP2 to combined final rescoring showed almost perfect agreement with Cohen's kappa value of 0.83.Follow-up FISH studies showed one amplified tumor.

**Conclusion:**

Our data support the need for finer granularity, classification, and understanding of HER2-low breast cancers. We also show that reproducibility between trained BP can be obtained, albeit with scoring at high power and low threshold for showing challenging interpretations.

## 1. Introduction

Since it was first described in 1984–1985, HER2 gene status evaluation and reporting criteria, and those of its surrogate marker the HER2 protein, have been an ever-evolving process. Most recently in 2022, largely driven by the results from the DESTINY B04 trial, HER2-low expression in invasive breast cancer was proposed as a separate diagnostic category [1]. HER2-low is currently defined as invasive breast carcinoma with HER2 immunohistochemistry (IHC) score of 1+ or 2+ with a negative in situ hybridization (ISH) assay [2]. Prior to that, HER2-nonamplified tumors were not considered eligible for any anti-HER2-targeted therapies. However, the DESTINY B04 trial demonstrated clinically significant and reproducible benefits to patients with primary and metastatic breast cancers and HER2-low expression using novel anti-HER2 antibody-drug conjugate trastuzumab deruxtecan [1]. This allows for the potential treatment of approximately 50% of all invasive mammary carcinomas [1]. However, many questions still remain to be answered, including is HER2-low a true separate biologic entity in mammary carcinomas. In this study, we identify the incidence of HER2 low in our institution and provide finer granularity into HER2 (1+) and HER2 (0) interpretation using a modified system for reporting and correlate our findings with HER2 fluorescence in-situ hybridization (FISH) status.

## 2. Materials and Methods

We retrospectively identified 114 invasive breast tumors comprised of core biopsies and surgical excision specimens of 112 patients (*n* = 114, including two patients with two tumors each) from the pathology database of Mayo Clinic, Jacksonville, FL, between January 2019 and August 2022, that were interpreted as HER2 negative. Two blinded BP independently reviewed the hematoxylin and eosin (H&E) and HER2 immunohistochemistry (IHC) slides. All HER2 slides contained an external positive (3+) on-slide control sample. Patients <18 years or >100 years and tumors with suboptimal HER2 control were excluded.

For HER2 IHC staining, the tissue was fixed in 10% neutral-buffered formalin, paraffin-embedded, cut at 4 microns on Superfrost Plus slides, air-dried, and placed in an oven at 60 degrees Celsius for 1 hour. The detection system used was Ventana ultraView Universal DAB, antigen retrieval CC1, and the antibody is Ventana Pathway HER-2/neu (clone 4B5), performed on the Ventana Benchmark Ultra instrument.

All 114 tumors were analyzed with dual-color *HER2* FISH assay, performed on 4-micron sections of formalin-fixed paraffin-embedded tissue using a probe specific to the chromosome 17q *HER2* locus (red fluorescent dye) and a copy number control probe recognizing centromere 17 (green fluorescent dye). *HER2*/centromere 17 ratio and *HER2*/cell ratio were established using signal quantification. HER2 FISH status was analyzed and reported in five groups (groups 1–5), according to the current CAP/ASCO guidelines [2]. According to this guideline, the definition of different groups is as follows: Group 1 (ISH positive): HER2-to-chromosome 17 centromere ratio ≥2.0, average HER2 copies ≥4.0; Group 2 (ISH positive): ratio ≥2.0, copies <4.0; Group 3 (ISH positive): ratio <2.0, copies ≥6.0; Group 4 (ISH equivocal): ratio <2.0, copies ≥4.0 and <6.0; and Group 5 (ISH negative): ratio <2.0, copies <4.0.

**Figure 1:**
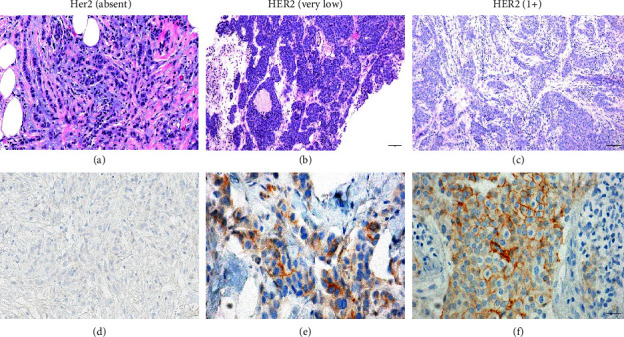
(a) H&E of a HER2 (absent) case (H&E slide, 100x magnification). (b) H&E of HER2 (very low) case (H&E slide, 100x magnification). (c) H&E of HER2 (1+) case (H&E slide, 100x magnification). (d) HER2 IHC of HER2 (absent) case (IHC slide, 400x magnification). (e) HER2 IHC of HER2 (very low) case (IHC slide, 400x magnification). (f) HER2 IHC of HER2 (1+) case (IHC slide, 200x magnification).

HER2 immunohistochemical slides were reviewed using 10x eyepiece with 20x objective (200x total magnification) and 10x eyepiece with 40x objective (400x magnification). The most recent 2018 CAP/ASCO HER2 scoring criteria were used in this study [2]. The HER2-negative category was subdivided into two categories for purposes of the study: HER2 (absent) (Figures 1(a) and 1(d)) and HER2 (very low), according to a reference study [3]. The following HER2 categories were used in this study and were defined as follows: HER2 (absent) (Figures 1(a) and 1(d)), complete lack of expression; HER2 (very low), >0% but <10% tumor cells with incomplete faint/barely perceptible membranous staining (Figures 1(b) and 1(e)); and HER2 (low, 1+), >10% faint incomplete membranous staining [3] (Figures 1(c) and 1(f)) **(**Table 1). Discordant cases between the two BP (A.N. and M.K.) were rescored together. Cohen's kappa test was used to analyze differences between the original final pathology report interpretations and rescored HER2 IHC results and between the two BP.

## 3. Results

Clinical data were provided for 114 tumors of 112 patients, summarized in Table 2 (Table 2). Among 114 tumors, 102 (89.5%) were core biopsies while 12 (10.5%) were surgical excision specimens. Patients were all women, whose age ranged from 39–96 years (mean 66.15, median 67). Tumors' laterality was nearly equally distributed, with 58 (50.9%) in the right breast while 56 (49.1%) were in the left with a mean tumor size of 0.85 cm and median size of 0.7 cm. Six women (5.3%) had two primaries (invasive lobular and invasive ductal carcinoma) in the same breast. Three patients (2.6%) had angiolymphatic invasion, and 5 (4.4%) had axillary lymph node metastasis at the time of surgery; 109 (97.3%) were alive, and 3 (2.7%) were deceased at the time of data collection completion (September 2022). There were 7 (6.1%) recurrences.

114 slides of 112 patients were reviewed. The initial scoring gathered from our medical record system, and the first round of rescoring and the final rescoring for HER2 at 200x and 400x magnification by breast pathologist 1 (BP1) and breast pathologist 2 (BP2) are summarized in Table 3 (Table 3). A comparison of the first round of rescoring between two pathologists showed substantial agreement with Cohen's kappa value of 0.67. Both comparisons of the first round of rescoring by BP1 and the first round of rescoring by BP2 to final combined rescoring showed almost perfect agreement with Cohen's kappa value of 0.83 whereas the comparison of initial scores and the final rescoring showed fair agreement with Cohen's kappa value of 0.35, related to the addition of HER2 (very low) category.

**Figure 2:**
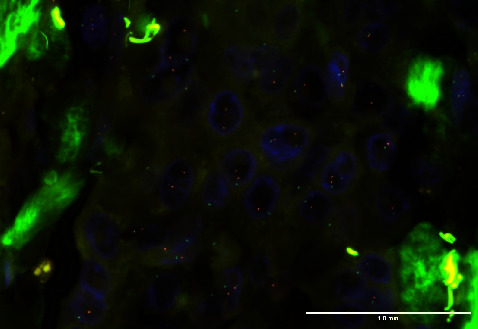
Fluorescence in situ hybridization of a HER2 (absent), ISH-negative case. Red signals localize to the *HER2* gene, while green signals represent the CEP17 locus (ISH slide, 400x magnification).

FISH results showed 111 tumors were FISH-negative Group 5 (Figure 2); 2 were FISH negative with comment Group 4; 1 was FISH-positive Group 1, with HER2 IHC Score (1+) with 60% membranous staining [2].

## 4. Discussion

Current guidelines state that HER2-amplified breast cancer can be targeted with anti-HER2 therapies such as trastuzumab [4]. However, traditional HER2-targeted therapy, or adjuvant trastuzumab, provides no benefit to patients with HER2-low tumors, as evidenced by the phase 3 NSABP-B47 trial by Fehrenbacher et al. [5]. As such, until recently, this subgroup of patients was treated according to HER2-nonamplified tumor protocols. Thus, evaluation and accurate classification of HER2 status have a critical role in the clinical management of breast cancer patients.

Current guidelines recommend simultaneously utilizing immunohistochemistry (IHC) and in situ hybridization to assess HER2 status in breast cancers [2]. Briefly, the established HER IHC interpretation categories are as follows: HER2 (0) (no membrane staining or membrane staining that is incomplete and faint/barely perceptible and in ≤10% of tumor cells) and HER2 (1+) (incomplete membrane staining that is faint/barely perceptible and in >10% of tumor cells) are categorized as HER2-negative tumors. HER2 (2+) (weak to moderate complete membrane staining observed in >10% of tumor cells) is categorized as equivocal and must be confirmed with in situ hybridization. HER2 (2+) with gene amplification and HER2 (3+) (circumferential membrane staining that is complete and intense and in >10% of tumor cells) tumors are defined as HER2-positive tumors and are eligible for targeted anti-HER2 therapies [2].

The DESTINY B04 trial alongside new and improved anti-HER2 armamentarium showed that even nonamplified breast tumors with low HER2 protein expression can benefit from novel anti-HER2 antibody-drug conjugates [6–8]. Trastuzumab deruxtecan was effective in targeting tumors with low HER2 expression by Modi et al. in a phase 1b trial [7]. According to the early results of this study, a consistent improvement in progression-free survival (PFS) was observed in HER2(1+) and HER2 (2+) and ISH-negative patients treated with trastuzumab deruxtecan. Banerji et al. [6] reported promising results with trastuzumab duocarmazine in HER2-low patients in their phase 1 trial. Another study by Denkert et al. [9] analyzed 4 different neoadjuvant trials in high-risk patients with early breast cancer. According to their results, patients who had HER2-low-positive tumors had significantly higher 3-year disease-free survival rates and 3-year overall survival rates compared to HER2 (0) tumors. Additionally, the DESTINY B04 trial for trastuzumab deruxtecan (Enhertu) showed significantly higher progression-free survival rates and overall survival rates in adult patients with unresectable or metastatic HER-low breast cancer who received a prior systemic therapy in the metastatic setting or developed disease recurrence during or within six months of completing adjuvant chemotherapy [1]. Furthermore, diverse combinational therapies are being studied for HER-low breast cancer treatment with promising preliminary results. Currently, there are several ongoing early clinical trials that are testing several diverse combinations of anti-HER2 agents with immunotherapeutics such as nivolumab, pembrolizumab, and durvalumab; CDK 4/6 inhibitors such as palbociclib; endocrine therapies and other combinations including chemotherapeutic agents and AKT inhibitors [10].

Although early on HER2-positive tumors were given a clinical emphasis and are widely studied, those represent a small fraction of all invasive mammary carcinomas. Recently, more emphasis has been placed on lower-grade tumors with aggressive biologic behavior. Even though HER2-low was mentioned in the literature as early as 2013, it was not until 2022 that this category of tumors gained interest. In part, this is due to the continued unmet clinical need in managing breast cancer, but also due to the major advances in HER2 therapeutics as described previously. Unfortunately, HER2-low in general as a subcategory within HER2-nonamplified tumors is still not well-studied and remains to be investigated. Notable disparities among pathologists still exist in classifying HER2 (0) and HER2 (1+) IHC scores [11, 12]. Due to promising results in the treatment of HER2-low tumors, HER2 status needs to be more accurately distinguished and defined. Furthermore, granularity into HER2 IHC scoring needs to be considered rather than binary reporting “positive” vs. “negative.” Reporting HER2 staining in more detail to include the percentage of staining in increments such as 5% or 10% and the type of staining (membranous, cytoplasmic, etc.) might be helpful to best determine the HER2 status and the treatment options of these patients. In our study, we reported HER2 rescoring to include the percentage of membranous staining to best determine the proper HER2 category for every patient.

In a recent study by Boyraz and Ly [3], the HER2-negative category was subdivided into 2: HER2 (absent) and HER2 (very low). According to this study, HER2 (absent) was defined as a complete lack of expression, and HER2 (very low) category was defined as incomplete faint/barely perceptible membranous staining up to 10% of tumor cells. The careful examination of HER2 (0) and HER2-low tumors (1+) under high magnification (200x and 400x) allowed for the recognition of more cases with HER2 expression. Notably, rescoring the HER2 (0) cases under 400x magnification resulted in identifying more cases with HER2 (very low) (33.3% of the group that previously reported as HER2 (0)) and HER2 (1+) (55.6% of the group that previously reported as HER2 (0)) expression.

We tested this approach in our study and recategorized our HER2 (0) patients to include HER2 (absent), HER2 (very low), and HER2 (1+) categories. Our results are comparable to Boyraz et al. from the 38 HER2 (0) tumors in our cohort, 14 (36,84%) were recategorized as HER2 (absent), 18 (47.38%) were recategorized as HER2 (very low), and 6 (15.78%) were recategorized as HER2 (1+). The use of high magnification during rescoring allowed us to identify faint membranous stainings that cannot be detected at lower magnifications, which is apparent from our previous results that demonstrate up-scoring of most of the HER2 (0) cases (*n* = 24, 63.15%) to HER2 (very low) or HER2 (1+). From the 76 cases that were initially scored as HER2 (1+), 1 of them was categorized as HER2 (absent), 17 of them were categorized as HER2 (very low), 57 of them were categorized as HER2 (1+), and one of them was categorized as HER2 (2+). These results indicate that there were no major changes in HER2 (1+) after rescoring, but with careful examination under high magnification, some of the HER2 (1+) tumors were rescored as HER2 (very low), which once again underscores the importance of evaluating HER2 staining under high magnification of 20x and 40x. The tumor that was rescored from HER2 (1+) to HER2 (absent) was found to have only cytoplasmic staining.

**Figure 3:**
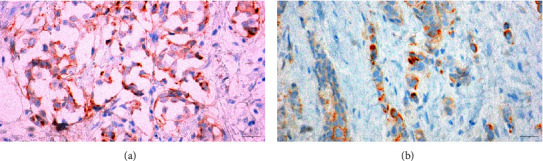
(a) H&E slide demonstrating cytoplasmic staining. (b) H&E slide demonstrating edge artifact.

Cytoplasmic staining is not scored in HER2 IHC interpretation; it may also be considered by some, perhaps, an artifact. However, it is not infrequent that nonmembranous staining, such as cytoplasmic staining or granular-type staining, is observed in standard HER2 IHC test interpretation. Traditionally, in binary HER2 IHC interpretation, breast pathologists might have assigned either a negative or indeterminate score and sent it for FISH. However, in the era of HER2-low, there are no established guidelines yet on how to manage such staining. Given the localization of the chromogen to the cytoplasm (Figure 3(a)) rather than the cellular membrane, it can be inferred that the staining is nonspecific. Edge artifact (Figure 3(b)), stronger staining at the periphery of the tissue, and no or less staining at the center, can be quite confounding. Multi-preanalytical and analytical variables can cause such artifacts and include fixation time and tissue thickness, to name a few. In our study, we found that such conundrums are best solved by reviewing with another breast pathologist or if needed, repeating the HER2 immunohistochemical stain.

It is of no surprise then that intraobserver and interobserver agreement rates in HER2 IHC test interpretation vary. Our data showed how Cohen's kappa agreements changed between the first and second rescoring by the two BPs. As evident from Cohen's kappa scores, agreement rates almost achieved the perfect agreement between two BP in the second rescoring, and the second rescoring gave the most accurate results, with better identification of the most accurate categories. The improvement in agreement rates also highlights the benefit of “pathologist training” in utilizing 20x and 40x objectives when evaluating HER2-low tumors. We emphasize the importance of focused training before interpreting and releasing HER2-low results in clinical practice.

The clinical significance of these new categories is currently still being investigated. Schettini et al. showed no statistically significant difference in overall survival between HER2-low and HER2-0 groups (*p*=0.787) [13], while Denkert et al. showed HER2-low tumors had a significantly lower pathologic complete response (pCR) rate than HER2-0 (29.2% vs 39.0%, *p*=0.0002) [9]. Per the authors, pCR was also significantly lower in HER2-low tumors vs. HER2-0 tumors in the hormone receptor-positive subgroup (17.5% vs 23.6%, *p*=0.024) but not in the hormone receptor-negative subgroup (50.1% vs 48.0%, *p*=0*·*21). Denkert also showed that patients with HER2-low tumors have significantly longer 3-year disease-free survival than did patients with HER2-0 tumors (83.4% vs. 76.1%) [9]. Furthermore, subtyping HER2-negative tumors also finds significance in other body systems and tumor types, such as those of p53-aberrant high-grade endometrial endometrioid carcinomas, where the authors suggest that tumors with HER2-very-low expression may behave less aggressively than HER2-low tumors (*p*=0.045) [14]. While these data are promising, current studies are still too limited to extrapolate significant conclusions, regarding the predictive and prognostic values of the proposed new HER2 categories.

Fluorescent in situ hybridization results were negative (Group 5) or negative with comment (Group 4) for most of the cases (113 tumors) in our study, which was an expected outcome for our low-HER2 expressing cohort. An unexpected result in our study was the ISH-amplified HER2 (HER2 immunohistochemical stain 1+) tumor. This tumor showed 60% weak incomplete membranous staining, yet also demonstrated an HER2/CEP17 ratio of 2.3 and HER2 signals/cell of 5.9, as such results could be interpreted as a “false-negative” HER2 IHC result. Reported false negative rates vary but have been around 4% for HER2 IHC-negative tumors with scores 0 and 1+ [15]. Our data show a much lower discordance rate of 0.9%, which could indicate differences in patient population, tumors tested, or improved detection methods.

## 5. Conclusion

Many studies are investigating low-HER2 expression in breast cancer, the associated clinicopathologic features, genetic profiles, and response to novel chemotherapeutics. Clinical guidelines, diagnostic criteria, and pathologic evaluation of HER2-low need further elucidation. Our results correlate with the literature and emphasize the importance of evaluating HER2 status at higher magnifications, especially for the HER2-low tumors where proper training in the evaluation may be warranted. HER2 scoring at higher magnifications such as 200x and 400x can be helpful in achieving more accurate scoring of HER2 status in breast cancer, which in term would allow for a personalized treatment plan.

## Figures and Tables

**Table 1:** Comparison of current criteria and study criteria in HER2 immunohistochemistry.

HER2 IHC score			
Current 2018 ASCO/CAP criteria	0		1+
	No staining or incomplete faint/barely perceptible membranous staining in up to 10% of tumor cells		Incomplete faint/barely perceptible membranous staining in >10% of tumor cells
Criteria that were used in this study	Absent	Very low	1+
	No staining observed	Incomplete faint/barely perceptible membranous staining in up to 10% of tumor cells	Incomplete faint/barely perceptible membranous staining in >10% of tumor cells

**Table 2:** Patient demographics and tumor characteristics of different rescored subgroups.

	HER2 (Absent)	HER2 (Very low)	HER2 (1+)	HER2 (2+)
Number of slides (*n*)	15	35	63	1
Age (mean)	65.93	63.71	67.50	76
Status (alive/deceased) (*n*)	14/1	35/0	61/2	1/0
Core biopsy/excision (*n*)	13/2	34/1	54/9	1/0
Type of tumor				
Ductal	10	22	42	1
Lobular	3	10	15	—
Poorly differentiated, not further categorized	2	1	2	—
Ductal + lobular	—	1	4	—
Ductal + metaplastic	—	1	—	—
Tumor size (mean)	7.85 mm	7.73 mm	8.81 mm	4 mm
Tumor grade (*n*)				
Grade 1	1	11	17	1
Grade 2	6	13	37	—
Grade 3	8	11	9	—
Hormonal status (*n*)				
ER+/PR+	6	25	56	1
ER+/PR−	3	3	4	—
ER−/PR+	1	3	2	—
ER−/PR−	5	4	1	—

**Table 3:** Recategorization of invasive breast cancer with HER2-negative and low (1+) protein expression into HER2 (absent), HER2 (very low), and HER2 low (1+) in 112 patients.

HER2 IHC score categories, initial	Initial scores per report, *n* and %	HER2 IHC score categories, rescoring	First rescoring BP1, *n*and %	First rescoring BP2, *n*and %	Second rescoring (BP1 and BP2), *n*and %
HER2 (0)	38 (33.3%)	HER2 (absent)	17 (14.9%)	20 (17.5%)	15 (13.2%)
		HER2 (very low)	31 (27.2%)	33 (28.9%)	35 (30.7%)
HER2 (1+)	76 (66.7%)	HER2 (1+)	64 (56.2%)	61 (53.5%)	63 (55.3%)
		HER2 (2+)	2 (1.8%)	NA	1 (0.9%)

## Data Availability

The data used to support the findings of this study are restricted by the Mayo Clinic Institutional Review Board in order to protect patient privacy.
